# Supplementary material for: Development of the fetal myocardium and changes in myocardial fibers orientation
Source: PLoS One. 2025 Apr 11;20(4):e0315691. doi: 10.1371/journal.pone.0315691 (PMC11990767; doi:10.1371/journal.pone.0315691)
Supplement: Attachment — Submitted filename: Plos One rebuttal letter rev 2.docx [file pone.0315691.s002.docx]

**Reviewer #1:**

The authors formulated the following 3 Aims:
1. "to assess in vivo the endocardial and epicardial longitudinal strain in fetuses of different gestational ages by STE as surrogate of layer maturation"
2. to confirm, on histological sections, the relative wall thickness of the endocardial and epicardial layers at different gestational ages.
3. to evaluate in silico the effect of different myocardial fiber arrangements on diastolic performance.

The endocardial and epicardial strain measurements using STE by their nature are very noisy. During early phases of gestation, when the heart is small the level of noise should be even higher. It is not clear what was the point of using strain as a measure of maturation given there are more common and more reliable measures of maturation such as ventricle volume, diameter or wall thickness.

*Thank you for your comment. STE suffers of some inter- and intra- observer variability. However, it is comparable/lower to other echocardiographic parameters. There are several papers demonstrating the feasibility of STE in fetal hearts, since 16 WoG. However, we stated some issues in the limitations (page 15, lines 430-436). On the other hand, strain imaging still remains an effective tool to study in vivo and not-invasively myocardial function. We reported common measures of heart maturation (page 9 lines 242-244 and figure 4), however, these parameters do not allow to study the fibers distribution. On the other hand, STE is a validated method to estimate fiber arrangement in the heart.*

Figure 2 and Table 1 contain overlapping information.

Table 1 is not necessary and should be omitted.

*Sorry, I didn’t understand. Figure 2 indicates longitudinal strain values by echo; table 1 is the fibers’ angles by histological measures.*

It is not clear what histological studies were intended to confirm. The criteria used to identify the boundaries between different layers are not described. Neither is clear how the fiber angle was measured and quantified in particular in situations illustrated in Figure 1,b3.

*We would like to thank the reviewer for raising the point related to the methodological aspects of histology which were not reported in the first version of the paper. We have specified how we proceeded for quantification and identification of the fiber orientations and angle evaluation in the material and methods session on page 7 and 8 on line 175-209. We added two reference on this topic, too.*

*We believe that the histological study was important for supporting the in silico modelling of different layers orientation to characterize the fiber orientation in the fetal myocardium and understand its impact on LV mechanics.*

Simulations utilize a 7-layer mechanical model, which is loosely connected to the rest of the study. It simulates pressure-volume curves which were not measured in this study. Reference 37 cited in the legend to figure 7 is not present in the list of references. It is not clear how the experimental values of fiber angle were determined. According to the methods sections histological analysis involved only 4 layers.

*Sorry for the mistake, we added ref. 37 (now 39) to the bibliography. The 7 layers model was used to create different models by changing fibers angulation. According to our and previous studies, the fiber angulation changes progressively from the endocardium to the epicardium. A 7 layers model was considered the most appropriate compromise between simplification of the model to allow data elaboration and adherence to in-vivo model. The angle of the single layer was attributed according to those found by histological study at the given gestational age. The models were tested by comparing the physiologic pressure-volume plots to that generated by the computational model in order to verify the goodness to fit. Figure 7 demonstrate the adequate adherence of the model to the physiological pressure-volume plot found in animal models and in neonates from previous studies.*

*In figure 8, we started from the 40GW arrangement and then we changed the fiber orientation to verify alternative setting and the role of alternative fibers arrangements on the pressure-volume plot.*

Overall the study is poorly designed and conclusions are not supported by the experimental information.

*Thank you for your comment. This is the first study facing the fetal development of myocardium in humans. To build an animal model in this setting is very difficult, and to obtain invasive data on healthy fetal heart is impossible, so we did the best (based on our knowledge) to confirm in vivo data with histological and computational models. Computational models are a validated alternative to animal model, at least to build an adequate hypothesis before to plan further and more invasive or ethical challenging studies.*

*We removed the last sentence of the conclusion, that might sound a little beat speculative.*

**Reviewer #2:**

This is an interesting paper that seeks to characterize the fiber orientation in the fetal myocardium and understand its impact on LV mechanics.

1. There are small grammatical issues throughout paper and abstract that should be addressed.

*Thank you for this comment. We reviewed the English language.*

2. Use of histological sections from fetal hearts. A portion of these were from fetal demise- do you know cause? While structurally normal heart development, issues with placenta and vascularization can affect myocardial development prior to fetal demise. This may affect some of your models.

*We would like to thank the reviewer for raising these important aspects, which we had considered in the selection of our cases but we did not specify in the first version. We excluded cases with intrauterine growth restriction and cases with maternal malperfusion vascularization. Moreover, according to fetal anthropometric indexes the selected hearts were within a normal range for the gestational weeks. We have specified it in the material and method session on page 6 lines 144-146.*

*As for the cause of intrauterine death of our cases: 4 were unknown, 1 for volvulus, 1 for uterine rupture, 3 for cordonal issues, 2 for infection; among the miscarriage 2 for cervical incontinence, 1 for early placenta abruption, 1 for superficial placental implantation.*

3. limitations related to angle dependence acknowledge. Are you able to use data from well characterized animal models with histology to validate the in silico model.

*Thank you for this suggestion. We agree that an animal model might improve the validation of in-silico model. Unfortunately, to build a fetal animal model is outside the purpose of this study, also because a very special ethical authorization should be needed to study fetuses of superior mammalians. In addition, before to build an animal model, a clear hypothesis should be presented to the ethical committee, so a pilot study is mandatory, and this study works in this direction.*

*Finally, the development of a similar animal study is too long to add those data to this paper.*
