# Supplementary material for: Development of the fetal myocardium and changes in myocardial fibers orientation
Source: PLoS One. 2025 Apr 11;20(4):e0315691. doi: 10.1371/journal.pone.0315691 (PMC11990767; doi:10.1371/journal.pone.0315691)
Supplement: Attachment — Submitted filename: Plos One Rebuttal Letter.docx [file pone.0315691.s003.docx]

***Reviewer #3 comments:***

- *This is revised manuscript. I did not review the paper in the first round. I believe that the major concerns raised in the previously reviews were only slightly addressed. The remaining concerns include the following:*
  - *1) Details of the histological analyses and results remain missing, as pointed out by the previous reviewer 1. Were the various layers identified? How were they identified? What were the thicknesses of the various layers?*

Thank you for your comment. We described more in detail in the Methods (lines from 152 to 186 – tracked changes file). In particular, we described how we computed the thickness of the layer and how we calculated the angle. The layers were distinguished according to the different orientation of the fibers, causing a different grade of orange (lighter in longitudinal fibers). In addition, we measured the angle of the fibers in the different layers. We specified that the myocardium was divided in seven layers to calculate the different angle of the fibers, similarly to Streeter et al. We divided the myocardium in 7 layers to have an adequate sample size for repeated measures of the fibers’ angle and to match the results found from histology with the in-silico model. In the results, we reported the values of wall thickness (from 1305 μm at 13 weeks of gestation to 5782 μm at 40 weeks of gestation) and the changes in fibers’ angulation in the seven layers (figure 6). The relative layer thickness was reported in Figure 5.

- *2) How (and what part of) the histological data was incorporated into the in silico calculations is not described or discussed. It seems the in silico calculation are performed without any specific regards for histological results (i.e. fiber orientations). Would it not be critical to incorporate these histological measures and see how this impacts on calculated function? I would expect that these calculations would require some assumptions about tissue compliance in the fiber direction but there is no description of this.*

Thank you for this question. We specified that in silico model was based on the histological data, in particular on the fibers’ orientation found in the different layers. Thus, the mesocardial thickness changed over the time, as well as the epicarial layer. On the other hand, endocardial layer remained unchanged because the relative wall thickness of that layer remained stable since the 13^th^ GW. We built a 7 layers model to merge at the best of our possibilities histological data and computational methods. In addition, data on LV volumes at different gestational ages were acquired from fetal echo. Data on elastic properties of myocardial fibers, and shear stress and pressure-volume plots data were available from previous studies (ref. 17-19 and 38-39) on humans or from animal fetuses. For ethical reasons, the acquisition of these data was not feasible in human fetuses. We already stated this point in the limitations (lines 379-384 tracked changed version). However, we believe that these data were adequate for our model, and the pressure-volume plots obtained from our model was in agreement with normal physiology of the LV.
