# Supplementary material for: Development of the fetal myocardium and changes in myocardial fibers orientation
Source: PLoS One. 2025 Apr 11;20(4):e0315691. doi: 10.1371/journal.pone.0315691 (PMC11990767; doi:10.1371/journal.pone.0315691)
Supplement: Attachment — Submitted filename: Rebuttal Letter Fetal heart Rev 5.docx [file pone.0315691.s005.docx]

*Review Comments to the Author*

*Reviewer #3: In my previous review I suggested a number of changes. A number of the suggested changes are inadequately addressed.

Strengths
The Abstract is much improved.


Remaining issues
1. The authors should better (more fully explain) how they aligned the histology measurements with the echo measurements.*

Thank you for this comment. We added Figure 7 to show the increase of epi/endo thickness over gestational age, and we added a statistical test to compare echo by histology.

Statistical analysis: “The correlation between epicardial to endocardial ratio assessed by speckle tracking and histology was performed by using Spearman’s Rank Test. Data were clusterized for gestational week. Thus, we calculated the mean echocardiographic epi/endo ratio and compared echo data with histological data at any gestational week.”

Results: “We found an excellent correlation between the epicardial to endocardial strain ratio and epicardial to endocardial wall thickness (r=0.950, p<0.001).”

*2. I still see little point in illustrating the data in Figure 8. Without a better description of what goes into the model and how you incorporated the histological data into the model, along with a careful discussion/explanation of the assumptions, I still think this data/analysis adds little of value to the paper. As a results and as I stated in my last review the basis for conclusions derived from these simulations remains a mystery.*

According with the Editor, we removed in-silico model section from our paper.

*Reviewer #4: The paper studied echo and histology of fetal hearts, and peformed diastolic pressurization on finite element model. There seems to several rounds of review by now. I try here to suggest mostly writing amendments.

1. There is some value in the data in terms of histology measurements for later stages of fetuses, which is lacking in the literature.
- However, the authors should detail the sample sizes of that investigation. How many samples were investigated for each age for both the thickness measurements and fiber orientation measurements?*

*- The authors provided r and p values for trend over age for thicknesses, but we typically expect a plot of meaurements over time and a best fit line across it. This is important to gain credibility and confidence in the conclusions.*

Thank you for this comment. We were able to collect about one patient for gestational week. Unfortunately, is difficult to have normal specimens eligible for this kind of study. This is the reason why figure 6 (now Figure 8) doesn’t show SD lines plot.

*- authors should also state clearly what was their crieteria for differentiating different layers from histology, it doesnt seem quite obvious from the histology images in supplementary figure. How did they choose which tissue is in which layer?*

Thank you for this comment. We further specified in the methods how we measured relative wall thickness. Of course, the contour might be irregular. Thus, multiple measurement were performed.

“The layers were identified according to different orientation of the fibers: vertically cut fibers of middle myocardium appeared lighter in color, because of the major amount of Orange G staining cytoplasm, making it possible to recognize them from the other layers. In the section it was therefore possible to clearly distinguish the longitudinal fibers of the endocardium and epicardium, colored purple red, and the fibers of the mesocardium, which presented a lighter shade as well as a different pattern. Using the same software, we were able to trace line segments for which the program gave us numerical values in micrometer (μm). We measured total wall thickness, non-compacted thickness, endocardial thickness, middle myocardium thickness and epicardial thickness. We carefully avoided artifacts. The measurements were repeated 5 times for each level and in different parts of the specimen. We recorded the mean value for each layer. In addition, the relative layer thickness was calculated.”

*- There is no statistics of trend over age for fiber orientation. This should be attempted if possible. If samples are too low for this, authors should state this. even low sample size data is valuable when there is no other data around.*

Thank you for this comment. We were able to build the plot available in figure 8, where the reader can see how the angle in the seven layers changes over the gestation. In particular, the epicardial layer is 90° oriented at 13 GW, the second layer (over 7) starts to be longitudinal at the 20^th^ GW, the third layer begins at 30 GW. Thus, we added this point in the limitations.

“Furthermore, the sample size was too small to develop myocardial fiber angulation plots for gestational age. Figure 7 simply represents the value obtained from the single sample at a given gestational age.”

*- The fiber orientation description in lines 231-232 is not true. the fibers dont go to + 90 or -90 according to figure 6. For a paper that focused on fiber orientation, not being able to characterize fiber orientation properly will be very negative to a reader/reviewer.*

Thank you for this comment. Figure 6 (now figure 8) represents a mean value. If we analyze the most endocardial myocytes, they are 90° oriented. This can be seen also in the ref (Streeter DD, Spotnitz HM, Patel DP, Ross J, Sonnenblick EH. Fiber orientation in the canine left ventricle during diastole and systole. Circ Res 1969;24(3):339–47.)

We better specified this point in the text.

“The angle of the fibers progressively decreased from 90 to 0 from the endocardium to the mesocardium and from 0 to -90° from the mesocardium to the epicardium. We divided myocardium in seven layers, in Figure 7 was shown the mean angle of the single layer. The angle gradient was stable in the endocardial layer, while was steeper in the epicardium at 13 weeks of gestation, reducing the angle gradient proportionally with the thickening of the epicardial layer.”

*2. The in silico work
- The model is not explained well. what software was used? FeBio? FEniCS? Abaqus? Ideally the simulation settings files should be shared for others to verify. The geometry assumed for the heart in the simulation is very consequential. it could complete change outcomes one way or another. How thick were the walls assumed in the simulations? are they realistic? from figure 7 they look very thin, might no be realistic. Otherwise, from experience, it should be very difficult to get those results in figure 7d, if we have a normal stiffness model and physiologic geometry.
- there should be limitations on how adult canine stiffness model is used here for fetal hearts. Should the fetal heart be less stiff?
- there should be limitation text on how active tension and systole was not simulated. For a proper comparison with echo, you need this, as including systolic behaviour can change comparisons with echo.
- I think there are several sweeping claims that should be removed:
a. line 273, claim that there is correlation between fiber orientation, histology and in vivo functional changes. The simulations did not account for active contraction, and contractility can change things. No formal correlation analysis is done.
b. line 277, histology shows improvement on LV function. how is this demonstrated? strains and most strain rates seems to decrease. How is improved function shown? histology cannot show function, the simualtions lacked active tension, and cannot prove that the histology should lead to increased function.
c. lines 350 - 355, claims that pressure volume plots with different fibers can explain histological changes in heart failure. Authors have not shown fiber orientation change in heart failure, and there can be a confusion between fetal heart investigation here, and heart failure in adult settings as it is. There is no investigation of stiffness, and so authors should not use stiffness to support claims here.*

According with the Editor, we removed this part of the paper.

*3. the echo measurements
- the measurements seem large from past reviews. but the variability is large (van Oostrum NH, European Journal of Obstetrics & Gynecology and Reproductive Biology. 2020 Jul 1;250:178-87.). The current measurements at the endo layer are beyond the higher end of that review. Given the high variability of measurements out there, this might be ok, but should be explicitly addressed in the text.*

We used a layer specific software; thus, the region of interest was smaller, this is the reason why the endocardial layer showed a slightly higher SD. We already stated this in limitations, however, we better specified this point.

“Thus, the standard deviation was higher than values calculated in full-thickness strain values.”

*- discussion section lines 303-310 seems overly optimistic on speckel tracking for echo strain meaurements. As the cited paper above showed, variability in hands of many different experts lead to highly varying strain results. I think the authors should acknowledge that there is a lot of variability out there, and use that to justify that their strains is higher than others.*

We partially agree on this. It’s true that layer specific strain is more complex. However, “global wall” strain is more robust, when acoustic window is adequate. As mentioned above, we added in the limitations the higher variability of layer specific strain.

*Finally: The title of the paper is about myofiber and LV mechanics, but the value of the paper seems to be more on the histology and echo measurements, less on the biomechanics and simulations, given that there's no active tension or systolic simulations. authors could conder address this.*

We agree that the title should change now: “Development of the Fetal Myocardium and Changes in Myocardial Fibers Orientation”
