# Supplementary material for: Development of the fetal myocardium and changes in myocardial fibers orientation
Source: PLoS One. 2025 Apr 11;20(4):e0315691. doi: 10.1371/journal.pone.0315691 (PMC11990767; doi:10.1371/journal.pone.0315691)
Supplement: Attachment — Submitted filename: Rebuttal letter PONE v. 6.docx [file pone.0315691.s006.docx]

Reviewer 3 has asked for minor changes in the manuscript.

The authors examine the developmental changes in LV myocardial structure (histology) and function (speckle tracking) during fetal development. The expected progressive changes in both structure and function were observed. The authors conclude that “Left ventricular myocardium maturation begins early during fetal life and starts from the differentiation of a subendocardial layer. The development of the epicardial layer is slower and it is completed late in fetal life.”

The paper has been improved and simplified by removal of the simulation data. I support publication provided the final adjustment are made.

1. I believe that the term “differentiation” that is used in the conclusion is not very informative. The conclusion would better read (and by more accurate) by stating: “As the fetus develops, both the relative tissue volume and peak systolic strain rates shift together from the endocardium towards the epicardium”.

*Thank you for the suggestion, we modified either in the abstract and in the conclusion.*

2. To measure strain, the myocardium was automatically divided into endocardial and epicardial layers.

What does the term “automatically” mean? Please provide details.

*Automatically means that it was not manually adjustable/modifiable. Echopac does not allow to modify the relative thickness of the layers, it just divides in inner and outer. Other software (i.e. Tomtec) permit to modify manually this parameter. Anyway, we specified what is manually modifiable and what not in methods.*

3. The authors state “All samples were included in paraffin, cut into 5 µm slices, and then stained with Azan-Mallory for measurement of layer thickness and myofibers orientation.” At this thickness, the term “all” implies thousands of slices. Do the authors mean that several transverse slices were examined from “all” the fetal hearts examined?

*Thank you for the comment. All might be misleading, we modified “The samples were included in paraffin…”. All means that all the samples were managed in the same way.*

4. The sentence lacks a verb. “As expected, wall thickness and diameters while the left ventricular volume increased based on a cubic regression model (Figure 5). “

*Sorry, probably, the sentence was accidentally truncated. The complete sentence was:*

*“As expected, wall thickness and diameters grew following a linear regression model, while the left ventricular volume increased based on a cubic regression model”*

5. The word “determinate” is an adjective not a verb. I think the word “suggests” might be considered.

Thank you, we changed accordingly.

6. The statement “These findings may be at least in part responsible of the lower tolerance to pre-load and after-load variation in preterm newborns.” I do not understand this conclusion.

*Several studies demonstrated that LV deformation properties are impaired in premature newborns compared to normal (at term) neonates. On the other hand, extreme premature newborns show a reduced tolerance to pre-load and after-load variations (most commonly seen in PDA – low systemic output for steal before, post ligation syndrome after closure, evidenced only in prematures and not in older infants), as well as a reduced response to inotropes. Based on our study, and on similar studies in premature newborns, these clinical evidence might be, at least in part, justified by an incomplete myocardial development in these subjects.*

Reviewer 4 has accepted the manuscript for publication but has dropped few suggestions that needs to be addressed. I thank the authors for responding well. Removing the simulations was a very good move. The focus on the echo and histology measurements is better now, and it covers a gap in the literature. I recommend publication of the article.

I just want to point out that the last paragraph of introduction stated the objective differently from the title of the paper. it stated quantifying thicknesses only, and did not mention fiber orientations.

*Thank you for the suggestion, we better specified in the aim this point.*

May I further suggest to the authors that one reason for the differential strains between epi and endo is likely due to the the curved surface of the heart and the radial expansion during systole. here's a reference to check out: Ren et al., Journal of the American Society of Echocardiography. 2023 May 1;36(5):543-52.

*We completely agree. Based on previous reviewers suggestions, we removed the in-silico model analysis, where, at least in part, we described this point. It is well known that strain depends not just on the relative angulations but also on the shape of the ventricle and on the curvature of the surface. Thus, we did not specified further in this paper. This is the reason why endocardial strain paradoxically decreases over the time (L/T ratio decreases over the time, in particular in the second trimester of gestation) and epi/endo strain ratio increases, accordingly with the epi/endo histologic relative wall thickness.*
